# The mild cognitive impairment window for optimal Alzheimer's disease intervention

**DOI:** 10.1177/25424823251370768

**Published:** 2025-08-18

**Authors:** Kevin Mekulu, Faisal Aqlan, Hui Yang

**Affiliations:** 1 Complex Systems Monitoring, Modeling and Control Laboratory, 8082 Pennsylvania State University, University Park, PA, USA; 2 Center for Human Systems Engineering, 5170 University of Louisville, Louisville, KY, USA

**Keywords:** Alzheimer's disease, biomarkers, delivery of health care, digital technology, early detection/diagnosis, public health

## Abstract

The FDA approval of disease-modifying Alzheimer's disease therapies marks a major shift in treatment but exposes a critical challenge: identifying patients during the mild cognitive impairment (MCI) stage when intervention is most effective. Despite early biological changes, most diagnoses occur after significant decline. Drawing from over 180 stakeholder interviews conducted through the NSF I-Corps program reveal major detection gaps across primary care, specialty access, and available tools. This commentary highlights the consequences of delayed diagnosis and proposes translational strategies to align early detection with therapeutic opportunity, positioning MCI as the critical window for Alzheimer's disease intervention.

## Introduction

The therapeutic landscape for Alzheimer's disease has undergone a fundamental transformation with the U.S. Food and Drug Administration's approval of monoclonal antibodies targeting amyloid-β pathology. These novel treatments, lecanemab and donanemab, mark the first interventions capable of modifying underlying disease processes rather than merely managing symptoms. However, this therapeutic breakthrough has exposed a critical vulnerability in our healthcare system: the inability to consistently identify patients during the mild cognitive impairment (MCI) stage, when these interventions demonstrate optimal efficacy. Despite compelling evidence that pathological processes begin decades before clinical manifestation, most patients receive a diagnosis only after substantial neurodegeneration and cognitive decline have occurred, often too late for maximum therapeutic benefit.

Through the National Science Foundation I-Corps program, we conducted over 180 comprehensive interviews with professionals across the dementia care spectrum, including neurologists, primary care physicians, neuropsychologists, speech language pathologists, geriatric psychiatrists, and administrators in various care environments. Interviews followed a structured discovery framework centered on identifying current workflows, clinical bottlenecks, and potential for digital innovation. Key insights were thematically grouped and used to shape the translational pathway proposed here. These discussions consistently highlighted the pressing need for earlier detection capabilities and identified substantial systemic barriers limiting our ability to recognize cognitive decline during this crucial intervention window. This perspective integrates these stakeholder insights to illustrate why early detection at the MCI stage constitutes both an urgent challenge and an unprecedented opportunity in contemporary Alzheimer's disease treatment.

## The shifting therapeutic landscape

The treatment paradigm for Alzheimer's disease has undergone a profound transformation from symptom management to disease modification. Clinical trials for lecanemab demonstrated a significant 27% reduction in cognitive decline compared to placebo, with notably stronger effects observed in patients with earlier-stage disease.^1^ Similarly, donanemab showed a 35% slowing of progression in early symptomatic Alzheimer's disease.^2^ Critically, post-hoc analyses from both studies suggest that intervention timing directly correlates with therapeutic benefit—an observation consistently emphasized by specialists in our stakeholder interviews.

“The greatest impact happens when patients begin treatment before substantial neuronal loss has occurred,” explained one neurologist. “Once significant atrophy is present, even the most promising treatments show diminished efficacy.” This perspective was echoed throughout specialist interviews, with another adding, “After decades of disappointment, we finally have treatments that can alter disease trajectory, but our detection capabilities haven't kept pace with these therapeutic advances.”

The biological rationale for this critical window is increasingly well-characterized. Amyloid accumulation begins approximately 15–20 years before clinical manifestation, followed by tau pathology, neurodegeneration, and eventual cognitive symptoms.^3^ The MCI stage, characterized by subtle cognitive changes that preserve functional independence, represents the earliest clinically identifiable phase of this continuum and the optimal intervention point for disease-modifying therapies. As one neurologist noted, “The battle against Alzheimer's will be determined by our ability to identify and treat patients during the MCI window.”

## The detection gap: causes and consequences

**Figure 1.**
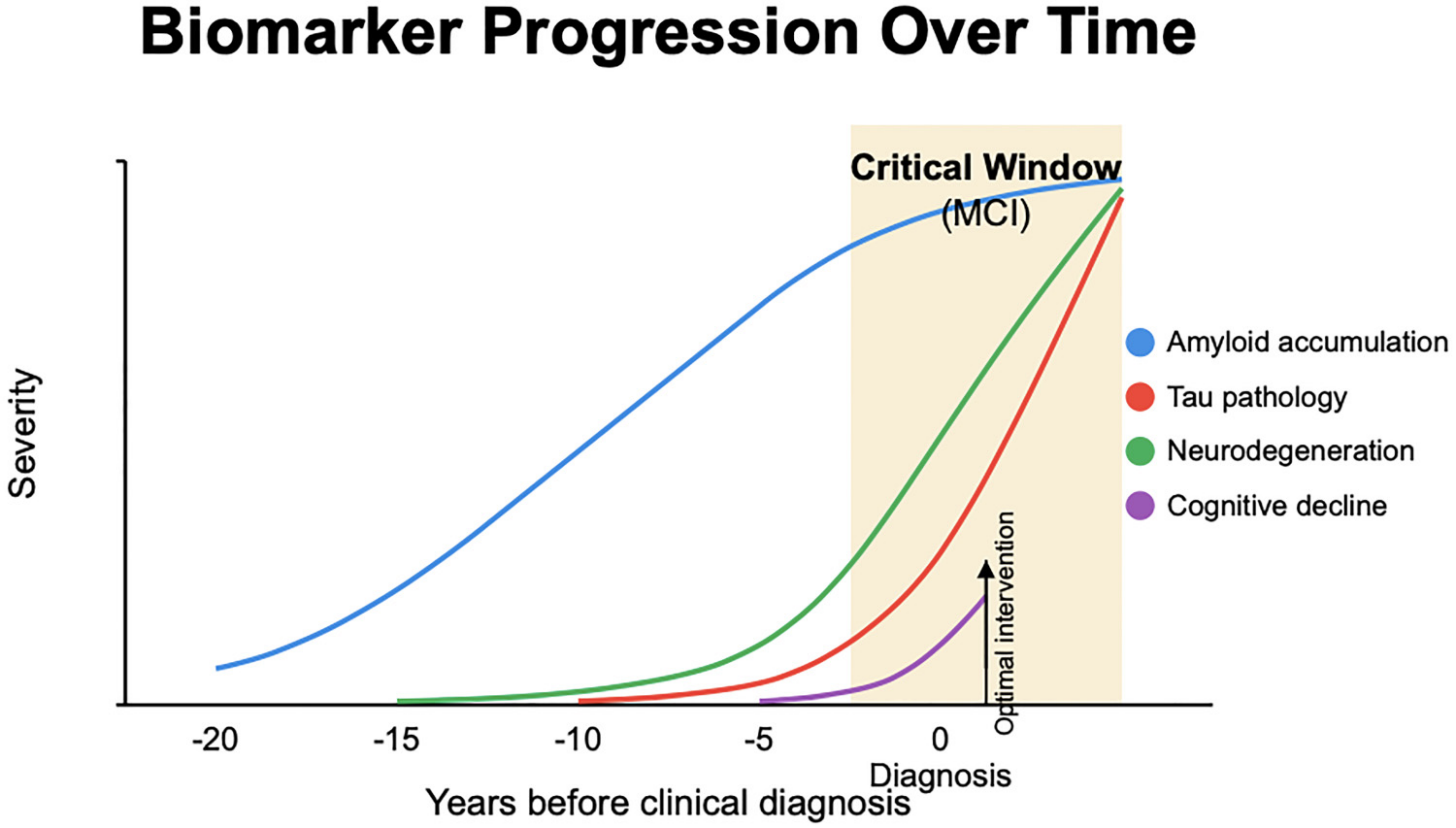
Timeline illustrating the “Critical Window” for optimal intervention in Alzheimer's disease. The graph shows amyloid accumulation beginning 15–20 years before clinical diagnosis, followed by tau pathology, neurodegeneration, and cognitive decline. The MCI stage (highlighted) represents the optimal intervention point for disease-modifying therapies, occurring before substantial neuronal loss but after biomarker changes enable accurate diagnosis.

The detection gap illustrated in Figure 1 between optimal intervention timing and current diagnostic practices stems from multiple interconnected factors revealed through our stakeholder interviews. Understanding these barriers is essential for developing effective solutions.

## Systemic barriers

Healthcare system constraints emerged as a primary obstacle to early detection. Primary care visits, typically lasting 15–20 min, must address multiple health concerns, preventive measures, and documentation requirements. “Cognitive assessment becomes a competing priority among many urgent needs,” explained one senior care administrator. Time constraints are particularly problematic because subtle cognitive changes require thorough evaluation beyond brief screening tools.

Knowledge gaps represent another significant barrier. “There is a serious knowledge gap at the primary care level in detecting early cognitive changes,” noted one primary care physician. “Distinguishing pathological decline from normal aging requires specialized expertise that isn't widely distributed in primary care.” This observation aligns with research showing that only 8% of primary care providers feel very confident in their ability to diagnose MCI.^4^

Access to specialty care creates additional complications. “Many patients face 6–12 month waiting periods for neuropsychological evaluation or specialty consultation,” reported one geriatric psychiatrist. These delays are particularly problematic given the progressive nature of Alzheimer's disease pathology, where each month represents potential irreversible neurodegeneration.

## Technical limitations

Current assessment tools demonstrate significant limitations for detecting early cognitive changes. Traditional instruments like the Mini-Mental State Examination and Montreal Cognitive Assessment were designed primarily to detect more advanced impairment, showing limited sensitivity for the subtle alterations characteristic of MCI. “Standard tools essentially miss the beginning of the disease process,” noted one neuropsychologist. “By the time patients screen positive, they've already progressed beyond the optimal intervention window.”

These instruments also suffer from significant practice effects with repeated administration, complicating longitudinal monitoring. “Patients remember the questions and improve their performance despite underlying decline,” explained one memory care administrator. This creates particular challenges in continuing care settings, where regular assessment is necessary but potentially misleading with current tools.

## Consequences of delayed detection

The human and economic consequences of this detection gap are profound. For patients, delayed diagnosis means irreversible neurodegeneration and cognitive decline that might have been prevented with earlier intervention. “Every month of delay represents brain cells lost forever,” emphasized one neurologist. This translates directly to diminished quality of life and increased caregiver burden.

The healthcare system faces significant costs from delayed detection. “Late-stage dementia care requires substantially more resources than early intervention,” noted one healthcare economist in our interviews. These increased costs stem from more intensive medical needs, safety concerns, and eventual residential care requirements. With disease-modifying treatments, this economic equation becomes even more compelling—early intervention may significantly reduce lifetime care costs despite treatment expenses.

Perhaps most concerning, the detection gap disproportionately affects vulnerable populations. “Patients with lower educational attainment, minority status, or rural residence face even greater barriers to early diagnosis,” observed one community health director. This creates potential for new treatments to exacerbate existing healthcare disparities unless detection capabilities improve across all care settings.

## Bridging the gap: a multi-faceted approach

Our stakeholder interviews revealed several promising strategies to improve early detection capabilities. Addressing this challenge requires coordinated efforts across multiple domains, from technological innovation to healthcare policy.

## Next-generation assessment tools

Digital biomarkers represent one of the most promising avenues for earlier detection.^5^ Speech analysis tools can identify subtle linguistic changes associated with early cognitive decline through automated assessment of semantic content, syntactic complexity, and acoustic features. “Natural language processing can detect patterns in speech that human evaluators might miss,” explained one speech technology researcher. These approaches offer particular promise for longitudinal monitoring, as they can be administered repeatedly without practice effects.

**Figure 2.**
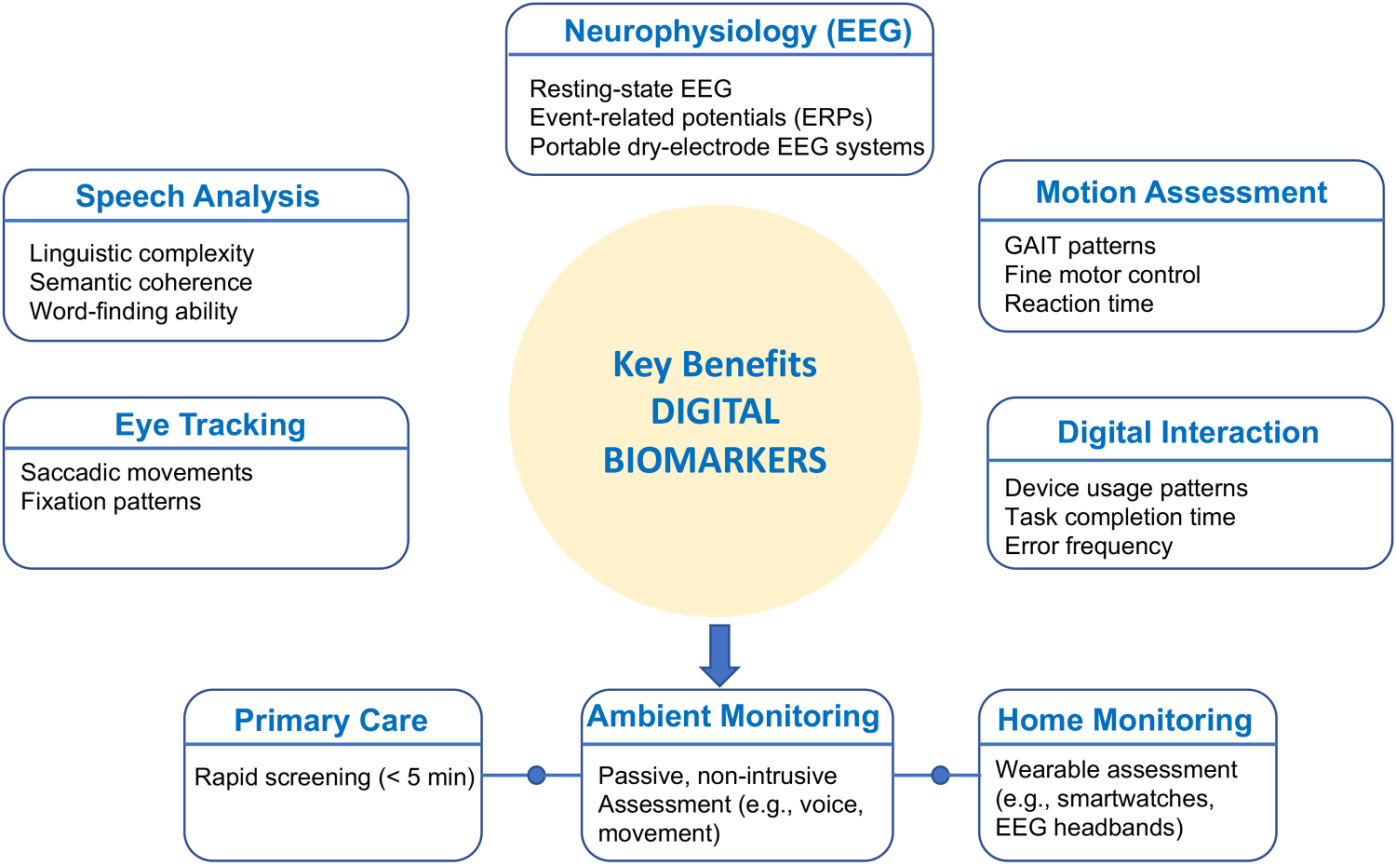
Overview of multi-modal digital biomarkers for early detection of cognitive decline. The diagram shows how speech analysis, eye tracking, motion assessment, neurophysiology (EEG), and digital interaction patterns can be integrated to provide comprehensive cognitive monitoring across primary care, specialty care, and home environments.

Other digital biomarkers showing promise include eye movement tracking, which can reveal attentional and executive function changes, and fine motor assessments through digital devices. “The way people interact with everyday technology can provide rich information about cognitive status,” noted one digital health specialist. These approaches, as shown in Figure 2, offer the possibility of passive, continuous monitoring without creating additional burden for patients or providers.

Neurophysiological markers, particularly electroencephalography (EEG), are increasingly recognized as powerful complements to behavioral digital biomarkers. Resting-state EEG and event-related potentials can provide direct insight into brain function and are sensitive to subtle cognitive changes associated with MCI. “EEG offers a more proximal measure of brain activity compared to speech or movement,” noted one researcher familiar with neurodiagnostic workflows. Importantly, recent advancements in portable, dry-electrode EEG systems have made real-world implementation more feasible, particularly in primary care and community settings. Incorporating these signals alongside behavioral data could improve diagnostic precision and enable earlier, more confident clinical decision-making.^6,7^

## Redesigning healthcare workflows

Beyond improved assessment tools, healthcare delivery systems must be restructured to facilitate earlier detection. Primary care workflow redesign emerged as a critical need from our stakeholder interviews. “We need assessment approaches that can be efficiently integrated into existing workflows,” noted one primary care physician. Potential adaptations include team-based screening protocols, where medical assistants or nurses administer initial assessments before physician evaluation and dedicated cognitive health visits covered by insurance.

Improved education and training for healthcare providers represent another essential element. “Many primary care providers lack confidence in their ability to detect and discuss cognitive changes,” explained one nursing professor with expertise in dementia care. Targeted training programs focused specifically on recognizing subtle cognitive alterations associated with MCI could significantly improve detection capabilities across care settings. These programs should emphasize distinguishing pathological changes from normal aging, an area where many providers report uncertainty.

Telemedicine and remote assessment capabilities offer particular promise for expanding access to specialty expertise. “Video-based cognitive assessments can extend specialized care to underserved areas,” noted an executive of a Telemedicine company. These approaches could help address the geographic disparities in access to dementia specialists while reducing wait times for evaluation.

## Policy and reimbursement considerations

Policy changes are necessary to support improved detection at the MCI stage. While CPT code 99483 provides reimbursement for comprehensive cognitive assessments,^8^ our interviews revealed it remains underutilized. “Many providers aren't aware of this code or how to efficiently implement the required documentation,” explained one billing specialist. Coupling this existing reimbursement pathway with streamlined digital assessment tools could transform early detection economics. Digital solutions that integrate with electronic health records and automatically generate appropriate documentation for 99483 requirements would address both clinical and financial barriers to implementation, making MCI screening more feasible in time-constrained settings. Beyond existing codes, regulatory pathways for novel digital biomarkers need clarification to accelerate their validation and adoption.

Public awareness campaigns represent another important policy initiative. “Many patients and families don't recognize the significance of early detection,” explained one public health director. Increased public understanding of the importance of early detection, particularly in the context of new disease-modifying treatments, could help reduce delays in seeking evaluation for early symptoms.

## The path forward: research priorities and practical next steps

### Research priorities

Our stakeholder interviews identified several critical research priorities to advance early detection capabilities. Differential diagnosis of dementia subtypes emerged as a paramount concern. “Current tools can identify cognitive impairment, but they can't reliably distinguish between Alzheimer's, vascular dementia, frontotemporal dementia, and other conditions,” emphasized one neurologist. This distinction is increasingly crucial as treatments become more targeted to specific pathologies. “With disease-specific treatments emerging, misdiagnosing the dementia type could mean missing the appropriate therapeutic window,” noted another specialist.

Longitudinal validation studies for digital biomarkers represent another top priority. “We need robust evidence that these technologies can detect meaningful cognitive changes over time,” emphasized one clinical researcher. These studies should track participants from cognitively normal status through MCI and beyond, correlating digital measures with traditional assessments, biomarker changes, and functional outcomes. Digital approaches that can distinguish patterns specific to different dementia types could revolutionize early detection and treatment selection.

Comparative effectiveness research represents another essential focus. “We don't yet know which combination of assessment approaches offers the optimal balance of sensitivity, specificity, and implementation feasibility,” noted one health services researcher. Studies directly comparing various screening methods across different care settings and populations could help identify the most effective strategies for widespread implementation.

Implementation science research is critical for translating promising approaches into practice. “Many innovations fail not because they don't work, but because they don't fit into real-world care environments,” explained one implementation specialist. Studies examining facilitators and barriers to adoption across diverse healthcare settings could help develop effective implementation strategies tailored to specific contexts.

Health equity research must be prioritized to ensure that improved detection capabilities benefit all populations equitably. “Without intentional focus on equity, new technologies risk exacerbating existing disparities,” cautioned one public health researcher. Studies examining how digital approaches perform across different demographic groups and strategies for ensuring equitable access are essential for preventing unintended consequences.

### Practical next steps

While research continues, several practical steps can begin immediately to improve early detection capabilities. Enhanced provider education represents a high-impact starting point. “Many providers lack specific training in recognizing subtle cognitive changes,” noted one professor of gerontology. Targeted continuing education programs focused on MCI detection could significantly improve identification rates even with existing assessment tools.

Clinical pathway development offers another immediate opportunity. “Standardized protocols for evaluation of subtle cognitive complaints could reduce inappropriate variation in care,” suggested one neurologist. These pathways should include specific guidance on when and how to conduct cognitive assessments, appropriate referral patterns, and communication approaches for discussing findings with patients and families.

Public awareness campaigns focused on the importance of early detection could help reduce delays in seeking evaluation. “Many people don't realize that what they perceive as ‘normal aging’ might be early signs of a treatable condition,” explained one medical researcher. These campaigns should emphasize the concrete benefits of early detection, particularly given the availability of disease-modifying treatments.

Cross-sector partnerships between healthcare systems, technology developers, research institutions, and patient advocacy organizations could accelerate progress. “No single entity can solve this challenge alone,” observed one healthcare administrator. Collaborative initiatives that leverage complementary expertise and resources offer the most promising path toward comprehensive solutions.

## Conclusions

The recent approval of disease-modifying Alzheimer's disease therapies has created both an unprecedented opportunity and an urgent challenge. Our extensive stakeholder interviews reveal a critical gap: while therapeutic intervention is most effective at the MCI stage, most patients are identified only after substantial progression. This gap stems from multiple factors, including time constraints in clinical encounters, limitations of traditional assessment tools, knowledge gaps among providers, and systemic barriers to specialty care access.

Emerging approaches, particularly digital biomarkers derived from speech, eye movements, fine motor control and neurophysiological signals, offer promising avenues for earlier and more sensitive detection. However, successful implementation will require coordinated efforts across multiple domains, from technological development to healthcare system adaptation and policy reform. The stakes could not be higher—closing this detection gap could fundamentally alter the trajectory of Alzheimer's disease for millions of patients worldwide.

As one neurologist poignantly noted in our interviews, “For the first time, we can offer patients more than just support through decline—we can offer hope for preserving cognitive function. But this hope depends entirely on our ability to identify the disease when intervention can still make a meaningful difference.” The critical window for optimal intervention in Alzheimer's disease is now within reach, but only if we can develop the detection capabilities to match our therapeutic advances.
